# DHRS2 inhibits cell growth and metastasis in ovarian cancer by downregulation of CHKα to disrupt choline metabolism

**DOI:** 10.1038/s41419-022-05291-w

**Published:** 2022-10-03

**Authors:** Zhenzhen Li, Yue Tan, Xiang Li, Jing Quan, Ann M. Bode, Ya Cao, Xiangjian Luo

**Affiliations:** 1 Key Laboratory of Carcinogenesis and Invasion, Chinese Ministry of Education, Department of Nuclear Medicine, Xiangya Hospital, Central South University, Changsha, Hunan 410078 PR China; 2 Cancer Research Institute, School of Basic Medicine, Central South University, Changsha, Hunan 410078 PR China; 3 Hengyang Medical College, University of South China, Hengyang, 421001 Hunan PR China; 4 Department of Pathology, Xiangya Hospital, Central South University, Changsha, Hunan 410078 PR China; 5 The Hormel Institute, University of Minnesota, Austin, MN 55912 USA; 6 Molecular Imaging Research Center of Central South University, Changsha, Hunan 410078 China; 7 Hunan Key Laboratory of Oncotarget Gene, Hunan Cancer Hospital and The Affiliated Cancer Hospital of Xiangya School of Medicine, Central South University, Changsha, Hunan 410078 China; 8 Key Laboratory of Biological Nanotechnology of National Health Commission, Central South University, Changsha, Hunan 410078 China; 9 National Clinical Research Center for Geriatric Disorders, Xiangya Hospital, Central South University, Changsha, 410078 China

**Keywords:** Cancer metabolism, Cell growth

## Abstract

The short-chain dehydrogenase/reductase (SDR) superfamily has essential roles in lipid metabolism and redox sensing. In recent years, accumulating evidence highlights the emerging association between SDR family enzymes and cancer. Dehydrogenase/reductase member 2(DHRS2) belongs to the NADH/NADPH-dependent SDR family, and extensively participates in the regulation of the proliferation, migration, and chemoresistance of cancer cells. However, the underlying mechanism has not been well defined. In the present study, we have demonstrated that DHRS2 inhibits the growth and metastasis of ovarian cancer (OC) cells in vitro and in vivo. Mechanistically, the combination of transcriptome and metabolome reveals an interruption of choline metabolism by DHRS2. DHRS2 post-transcriptionally downregulates choline kinase α (CHKα) to inhibit AKT signaling activation and reduce phosphorylcholine (PC)/glycerophosphorylcholine (GPC) ratio, impeding choline metabolism reprogramming in OC. These actions mainly account for the tumor-suppressive role of DHRS2 in OC. Overall, our findings establish the mechanistic connection among metabolic enzymes, metabolites, and the malignant phenotype of cancer cells. This could result in further development of novel pharmacological tools against OC by the induction of DHRS2 to disrupt the choline metabolic pathway.

## Introduction

Globally, ovarian cancer (OC) represents the seventh leading cause of cancer mortality in women [1]. Approximately 70% of ovarian cancers are diagnosed at an advanced stage, including with development of intra-abdominal metastatic colonization [2, 3]. Unfortunately, the 5-year survival rate for OC is less than 35% [4–6].

The short-chain dehydrogenase/reductase (SDR) superfamily has essential roles in lipid metabolism and redox sensing [7]. The family members can function as oxidoreductases, lyases, and isomerases to catalyze substrates, including steroids, retinoids, lipids, polyols, ribonucleotides, and xenobiotics [8–10]. Thus, they can determine the switch between active and inactive states of these signaling molecules. Dehydrogenase/reductase member 2 (DHRS2), also known as Hep27, belongs to the NADH/NADPH-dependent SDR family [11].

In recent years, accumulating evidence highlights the emerging association between SDR family enzymes and cancer [12–15]. The *DHRS2* gene is located on chromosome 14q11.2, and deletion in this region occurs in a variety of cancers, suggesting that DHRS2 might play an indispensable role in the incidence and progression of cancer [16]. Reduced expression of DHRS2 has been found in various tumor tissues compared to the normal counterparts. The change in expression contributes to the increased proliferation, migration, and chemoresistance of cancer cells [11, 17–20]. DHRS2 is markedly downregulated in esophageal squamous cell carcinoma (ESCC) and negatively correlates with tumor invasion, lymph node metastasis, and clinical stage [17]. Forced DHRS2 expression decreases cellular ROS levels and the ratio of nicotinamide adenine dinucleotide phosphate oxidation/reduction (NADP/NADPH). It also inhibits the p38 MAPK pathway and MMP2 expression to hinder ECSS cell growth and motility [17]. In addition, DHRS2 re-expression inhibits cell proliferation by interrupting intracellular lipid homeostasis in nasopharyngeal carcinoma [18]. It has been reported that treatment with histone deacetylase inhibitors (HDACi) upregulated DHRS2 expressions, and high expression of DHRS2 sensitized OC cells to HDACi treatment [21]. Whereas, how DHRS2 functions in OC has not been fully clarified.

Choline metabolism is an important part of intracellular phospholipid metabolism [22]. Choline kinase α (CHKα) is the first rate-limiting enzyme in choline metabolism that catalyzes choline phosphorylation to yield phosphorylcholine (PC) in the phosphatidylcholine (PtdCho) biosynthetic process [23]. PtdCho exerts a dual role as the most abundant structural phospholipid component in eukaryotic cell membrane systems, and also as a substrate to generate lipid second messengers, including phosphatidic acid and diacylglycerol [24]. The “cholinic phenotype” is characterized by increased PC and total choline-containing compounds (tCho), and has been observed in multiple cancers, especially brain cancer and OC [22, 25]. Elevated activity of choline-metabolic enzymes, such as PtdCho-specific phospholipase D (PC-PLD) and phospholipase C (PC-PLC) have been observed in OC cells [25, 26], indicating high activity of choline metabolic pathways. Studies have shown that high PC and low glycerophosphorylcholine (GPC) levels promote the malignant transformation of epithelial ovarian cells [25].

Here, we demonstrated that DHRS2 inhibited the growth and metastasis of OC cells. Mechanistically, CHKα promoted the activation of downstream AKT signaling and choline metabolism. DHRS2 downregulated CHKα expression in a post-transcriptional manner. Downregulation of CHKα mediated the tumor-suppressive role of DHRS2 in OC through the interruption of choline metabolism. The present study establishes mechanistic connections among metabolic enzymes, metabolites, and the malignant phenotype of cancer cells, and further develops novel pharmacological tools against OC by the induction of DHRS2 and targeting the CHKα/choline metabolic pathway.

## Materials and methods

### Cell culture

OC OVCAR3 (ATCC HTB-161), SKOV3 (ATCC HTB-77), HO-8910, and PEO1 cells were grown in RPMI 1640 media supplemented with 10% v/v heat-inactivated fetal bovine serum (Invitrogen, Carlsbad, CA, USA), 1% w/v glutamine and 1% w/v antibiotics. All the cell lines involved were cultured at 37 °C in a humidified incubator containing 5% CO_2_. Cells were routinely tested for mycoplasma using MycoBlue Mycoplasma Detector kit (Vazyme). Shanghai Biowing Applied Biotechnology (Shanghai, China) characterized short tandem repeat (STR) profiling to confirm the cell lines authenticity.

### Reagents and antibodies

Choline and Actinomycin D were acquired from MedChemExpress (MCE, Princeton, NJ, USA). Oil red O was purchased from Sigma-Aldrich (St. Louis, MO, USA). The antibodies against β-actin (sc-8432,1:5000) and perilipin1 (sc-390169, 1:500) were purchased from Santa Cruz Biotechnology (Santa Cruz, CA, USA). The anti-DHRS2 (PA5-25258, 1:1000) was from Thermo Fisher Scientific (Thermo Scientific, MA, USA). The antibodies to detect CHKα (13520-1-AP, 1:500), AKT (10176-2-AP, 1:1000), and phospho-AKT (S473) (66444-1-Ig, 1:1000) were purchased from Proteintech (Rosemont, IL, USA). The antibody against Ki67 (ZM-0166) was obtained from ZSGB-BIO (Beijing, China).

### RNA sequencing

Total RNA was extracted from OVCAR3-CON (*n* = 3) or OVCAR3-DHRS2 (*n* = 3) cells. RNA samples were sequenced by using Illumina NovaseqTM 6000 platform to generate raw data. RNAs differential expression analysis was performed by edgeR software between two different group and two different samples. The genes/transcripts with the parameter of *p* < 0.05 and fold change >2 or fold change <0.5 were considered differentially expressed genes/transcripts. GO Enrichment and KEGG pathway analyses were used for functional pathway analysis.

### UPLC-MS/MS analysis

Metabolites were detected using QTRAP® 6500+ UPLC-MS/MS System (AB SCIEX, USA). UPLC separation was performed using a reverse-phase column (Luna® 200A HILIC, 2.0 mm × 100 mm × 3.0 µm, Phenomenex, USA) with a binary gradient solvent system at a flow rate of 0.25 Ml/min: mobile phase A (pH = 9.0: 95% (vol/vol) water, 5% (vol/vol) acetonitrile, 20 mM ammonium hydroxide, 20 mM ammonium acetate), mobile phase B (100% acetonitrile). The injection volume was 2 μL and the total run time for each sample was 23 min: 85% (vol/vol) B 0.0 min; 85% B to 30% B, 3.0 min; 30% B to 5% B, 12.0 min; 5% B, 15.0 min; 5% B to 85% B, 16.0 min; 85%, 23.0 min. In the positive/negative ion mode, ionization was performed using the following parameters: the ion spray temperature 500 °C. Collisionally activated dissociation (CAD) gas: medium level. Curtain gas 35–40 psi; ion source gases 1 and 2:45 psi; Declustering potential: + 93 in positive ion mode/−93 in negative ion mode; Entrance potential: +10 in positive ion mode/−10 in negative ion mode; Collision cell exit potential: +10 in positive ion mode/−10 in negative ion mode. Metabolites were detected in the MRM mode using optimized *m*/*z* values of precursor ions (Q1) and product ions (Q3) and collision energies. Multiquant^TM^ 3.0.2 software was used for data pre-processing analysis. Principal component analysis (PCA) and pathway analysis were performed on the data by MetaboAnalyst 5.0.

### Oil red O staining

Cells were seeded in a six-well plate. The next day the media were discarded and the cells were fixed with methanol for 10 min. This was followed by staining with the diluted oil red O solution for 30 min. Cells were washed with PBS three times, and then observed under an optical microscope.

### Three-dimensional (3D) invasion assay

3D cell culture was performed as described previously [27]. Matrigel (20%; Corning, New York, USA) was added to the 24-well plate, placed in a 37 °C incubator for 60 min, and then washed twice with serum-free medium. Cells (2 × 10^3^) resuspended in 200 μl of 10% Matrigel solution were added to each well, and allowed to stand for 30 min in a 37° incubator. Then 200 μl of complete medium were added to the 24-well plate. On the 3rd, 5th, and 7th days, the proportion of spheroids or the number of pseudopodia was observed by microscope.

### RNA immunoprecipitation (RIP) assay

The RIP assay was performed using a MagnaRIP RNA-Binding Protein Immunoprecipitation kit (Millipore) according to the manufacturer’s protocols. 4 μg of control IgG antibody (1:20) or anti-DHRS2 was bound to magnetic beads and incubated with the corresponding cell lysates at 4 °C overnight. Next, proteinase K was used to digest the protein, and TRNzol (TIANGEN, China) was applied to extract the RNA. Finally, reverse transcription and qRT–PCR were performed to determine the *CHKα* mRNA level.

### RNA stability assay

Cells were incubated with actinomycin D at a concentration of 5 μg/ml, and then were collected after 0 h, 4 h, 8 h, or 12 h. Cellular RNA was extracted and analyzed by q-PCR.

### Micro positron emission tomography (PET)/computed tomography (CT) imaging

Mice were subjected to micro-PET/CT analysis when the subcutaneous transplanted tumor reached to an appropriate volume. ^11^C-choline (37 MBq) was injected intravenously to mice after anesthesia inhalation with isoflurane gas. After absorption for 10 min, images were acquired by the micro-PET scanner (Mediso, Hungary). Proper body temperature was maintained using a heating pad throughout the imaging procedures. Standardized choline uptake values were acquired as dividing the regions of interest concentration by the ratio of the injected activity to the body weight.

### Animal studies in nude mice

Animal care experimental procedures were performed in accordance with the approval of Xiangya Hospital of Central South University (Changsha, China). The sample size for animal experiments was estimated according to the type of study design and the purpose of the experiment. Animal experiments were conducted using randomized groups, and the investigator was blinded to the group allocation during the experiment. To evaluate the effect of DHRS2 on OC growth in vivo, 5-week-old female BALB/c nu/nu mice (*n* = 6 for each group) were subcutaneously injected with OVCAR3-CON or OVCAR3-DHRS2 cells (5 × 10^6^). Tumor volume was calculated according to the formula (*V* = length × width^2^/2), and measured every other day. At the end of experiments, the mice were euthanized by CO_2_ inhalation and the tumors were stripped and weighed.

To explore the role of DHRS2 in the metastasis of OC, OVCAR3-CON or OVCAR3-DHRS2 cells (5 × 10^6^) were injected into the abdominal tissue of BALB/c nude mice (*n* = 6 for each group) to construct mice abdominal cavity metastasis model. Mice were weighed once every 2 days. After 45 days, the tumor intraperitoneal metastasis was observed in the mice.

### Statistical analysis

Statistical analyses were performed using the two-tailed Student *t*-test. Statistical analysis showed mean and standard deviation (error bars) of the replicate means. A *p*-value of <0.05 was considered statistically significant. GraphPad Prism V8.0 software program (GraphPad Software, La Jolla, CA, USA) was used for most of the statistical analyses.

## Results

### DHRS2 inhibits the growth and invasion of OC cells

**Fig. 1:**
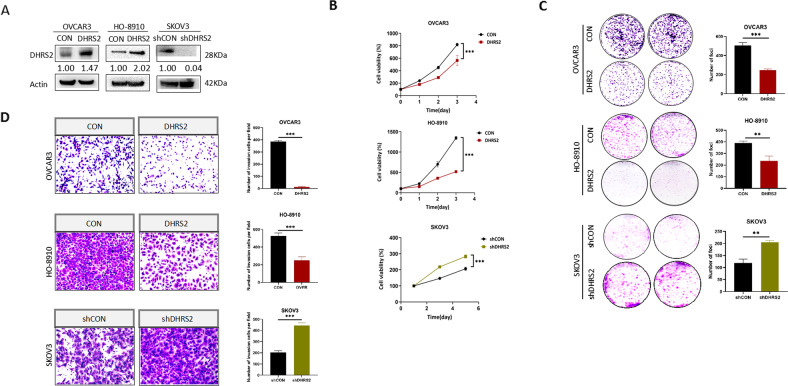
DHRS2 Inhibits the growth and invasion in ovarian cancer cells. **A** DHRS2 protein expression levels of in OVCAR3-CON, OVCAR3-DHRS2, HO-8910-CON, HO-8910-DHRS2, SKOV3-shCON, and SKOV3-shDHRS2 cells. **B** Cell viability of the designated groups was detected by MTS assay. **C** Foci formation ability of the designated groups was determined by colony formation assay. **D** Cells were seeded in the upper chamber with Matrigel coating for 72 h, and cell invasion ability of the designated group was determined. Data are shown as mean values ± S.D. of independent, triplicate experiments. The asterisks (*,**,***) indicate significant differences (*p* < 0.05, *p* < 0.01, *p* < 0.001, respectively).

To gain insight into the role of DHRS2 in ovarian cancer (OC), we first measured DHRS2 expression in a variety of OC cell types. We found that the protein level of DHRS2 in most OC cell lines is low, whereas it is relatively high in SKOV3 cells (Supplementary Fig. 1). Thus, we established stable OVCAR3-DHRS2 and HO-8910-DHRS2 cell lines by transfection of a DHRS2-expression vector into OVCAR3 and HO-8910 cells, respectively. We also established a stable knockdown SKOV3-shDHRS2 cell line by using short hairpin RNA(shRNA) targeting DHRS2 (Fig. 1A). Results of cell growth and foci formation assays showed that forced DHRS2 expression significantly suppressed the growth of both OVCAR3 and HO-8910 cells (Fig. 1B, C). Conversely, knockdown of DHRS2 substantially increased the tumorigenicity of SKOV3 cells. Next, we explored whether DHRS2 could affect the invasive capability of OC cells. Cell invasion assay results demonstrated that re-expression of DHRS2 dramatically reduced the number of cells invading through Matrigel, whereas DHRS2 knockdown enhanced OC cell invasion (Fig. 1D). These results indicate that DHRS2 inhibits the growth and invasion of OC cells.

### A combination of transcriptome and metabolome analysis reveals the interruption of choline metabolism by DHRS2

**Fig. 2:**
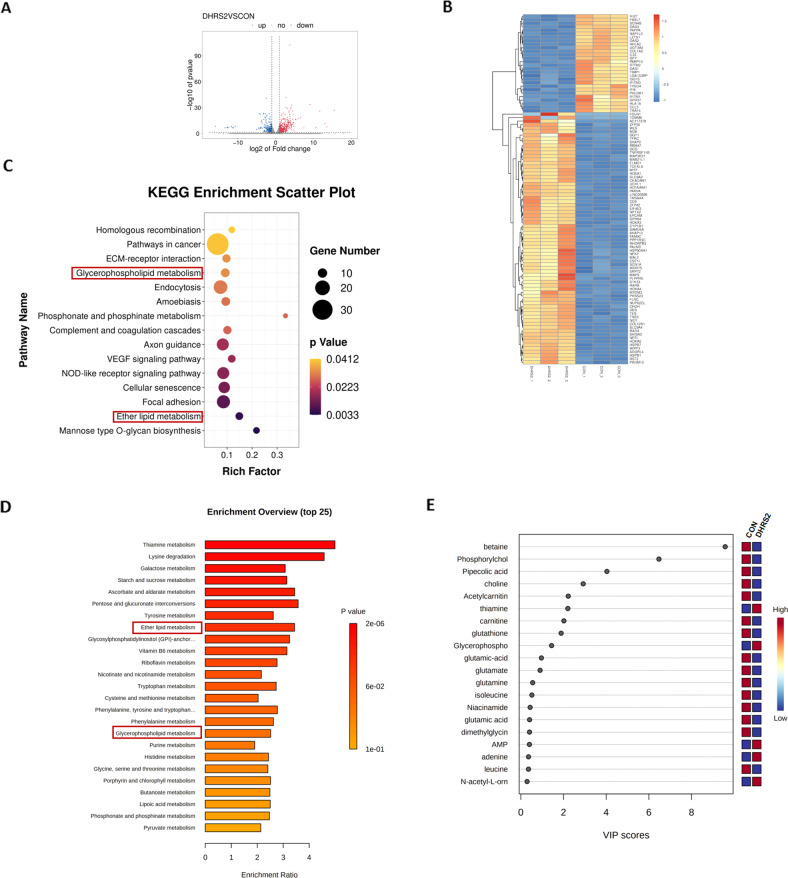
Combination of transcriptome and metabolome reveals the interruption of choline metabolism by DHRS2. **A** Scatter plots of all expressed genes in OVCAR3-CON and OVCAR3-DHRS2 cells. Blue color indicates upregulated genes, red indicates downregulated genes, and gray indicates non-regulated genes. The ‘regulated gene’ is defined as a gene with FDR ≤ 0.001 and abs (log2(Y/X)) ≥ 1. **B** Heatmap of the transcriptome changes in OVCAR3-CON and OVCAR3-DHRS2 cells (*n* = 3 for each group). **C** Enriched KEGG pathway analysis in the transcriptome of OVCAR3-CON and OVCAR3-DHRS2 cells. **D** Pathway enrichment analysis in the metabolome of OVCAR3-CON and OVCAR3-DHRS2 cells. **E** Important features identified by partial least squares-discriminant analysis (PLS-DA). The colored boxes on the right indicate the relative concentrations of the corresponding metabolite in OVCAR3-CON and OVCAR3-DHRS2 cells.

In order to interrogate the underlying mechanism of DHRS2-induced inhibition of cell growth and invasion, DHRS2-transfected OVCAR3 and vector control cells were used for RNA sequencing (RNA-Seq) and metabolome experiments. For the RNA-seq approach, 730 upregulated and 367 downregulated genes were identified by differentially expressed gene (DEG) analysis of DHRS2-transfected cells compared to control cells (Fig. 2A, B). KEGG pathway enrichment further illustrated that of the DEGs with significance, 16 genes were annotated and enriched in ether lipid or glycerophospholipid metabolism pathways, including *PLD1, PLA2G4A, CEPT1, PLPP2*, and *ACHE* (Fig. 2C). For the metabolome approach, 155 upregulated and 153 downregulated metabolites were identified in DHRS2-transfected cells relative to the vector control. Pathway enrichment analyses showed that ether lipid and glycerophospholipid metabolic pathways were also among the top 25 significant enrichment pathways (Fig. 2D), which is in accordance with the result of transcriptome analysis. Of the top 20 differentially expressed metabolites, glycerophosphocholine (GPC) was substantially upregulated; and phosphorylcholine (PC) and choline were downregulated (Fig. 2E). These metabolites all converge on choline metabolism. Thus, combined transcriptome and metabolome analyses provide a clue showing that the interruption of choline metabolism might be involved in DHRS2-induced inhibition of OC progression.

### CHKα downregulation is involved in the interruption of choline metabolism by DHRS2

**Fig. 3:**
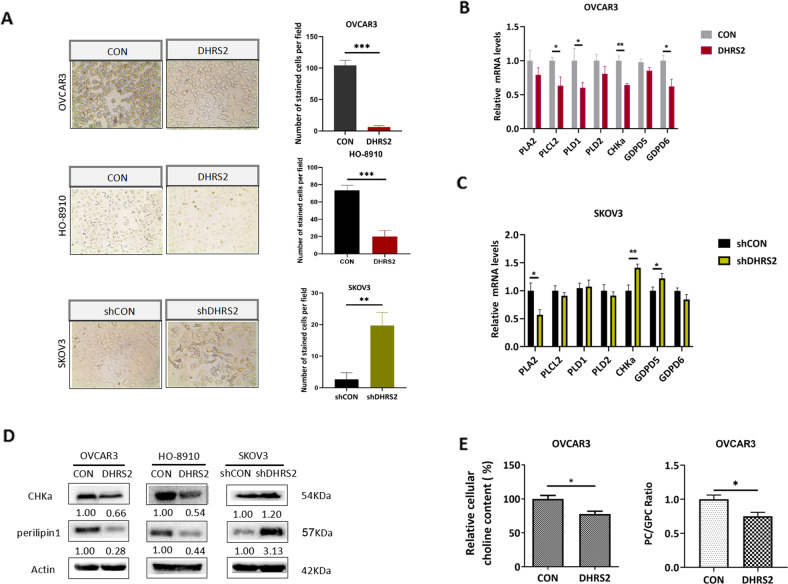
CHKα downregulation is involved in the interruption of choline metabolism by DHRS2. **A** Oil red O staining indicating the content of LD in OVCAR3-CON, OVCAR3-DHRS2, HO-8910-CON, HO-8910-DHRS2, SKOV3-shCON, and SKOV3-shDHRS2 cells. The mRNA levels of PLA2, PLCL2, PLD1/2, CHKα, and GDPD5/6 in **B** OVCAR3-CON, OVCAR3-DHRS2 and **C** SKOV3-shCON and SKOV3-shDHRS2 cells detected by RT-qPCR. **D** The protein expression levels of CHKα and perilipin1 in OVCAR3-CON, OVCAR3-DHRS2, HO-8910-CON, HO-8910-DHRS2, SKOV3-shCON, and SKOV3-shDHRS2 cells. β-actin was used as a loading control. **E** The cellular choline content and PC/GPC ratio in OVCAR3-CON and OVCAR3-DHRS2 cells. Data are shown as mean values ± S.D. of independent, triplicate experiments. The asterisks (*,**,***) indicate significant differences (*p* < 0.05, *p* < 0.01, *p* < 0.001, respectively).

Phosphatidylcholine (PtdCho), an important product of choline metabolism, represents the main membrane phospholipid component of lipid droplets (LD), and affects LD proliferation and division. We used oil red-O staining to examine the change of LD content induced by DHRS2. The results demonstrated a significant decrease of LD accumulation in DHRS2-transfected OVCAR3 and HO-8910-CON cells, whereas an enhanced LD content in SKOV3-shDHRS2 cells was observed (Fig. 3A). This result further supports the idea that DHRS2 hampers choline metabolism in OC cells.

Next, the transcriptional profile of genes involved in the choline metabolic pathway was determined in DHRS2-transfected and vector control cells. DHRS2 overexpression exhibited inhibitory effects on mRNA levels of the enzymes associated with choline metabolism, including *PLA2*, *PLCL2, PLD1, CHKα, GDPD5*, and *GDPD6*. In contrast, DHRS2 knockdown upregulated the transcriptional level of *CHKα* and *GDPD5* (Fig. 3B, C). Among these enzymes, CHKα mRNA showed the most dramatic change induced by DHRS2. Moreover, the alteration of CHKα protein expression was in agreement with that of the mRNA level, that is, CHKα expression inversely correlated with that of DHRS2 (Fig. 3D). In the choline metabolic pathway, CHKα is responsible for converting choline into PC. Accumulating evidence shows that increased PC content, and especially the PC/GPC ratio, boosts the proliferation and invasion of cancer cells. We observed that overexpression of DHRS2 inhibited the protein expression of perilipin1, an LD coat protein, and vice versa (Fig. 3D). Moreover, forced expression of DHRS2 substantially downregulated the PC/GPC ratio in OC cells (Fig. 3E). Thus, we conclude that the downregulation of CHKα is involved in the interruption of choline metabolism by DHRS2.

### Downregulation of CHKα mediates the inhibitory effect of DHRS2 on the growth and invasion of OC cells

**Fig. 4:**
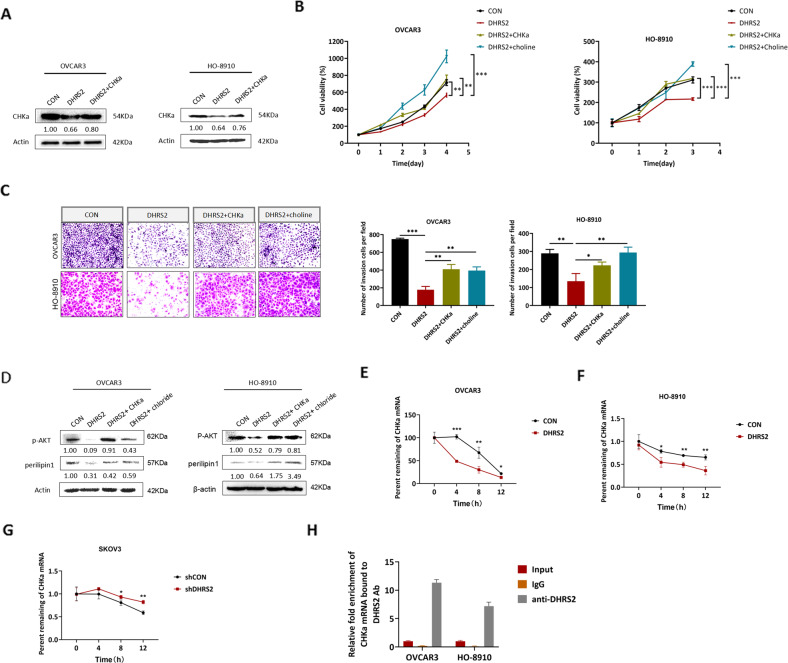
Downregulation of CHKα mediates the inhibitory effect of DHRS2 on the growth and invasion of OC cells. **A** The protein expression levels of CHKα in the designated group. After treated with 10 μM choline for 3 days or transfection with CHKα, **B** the viability of the cells was detected by MTS, **C** the invasive capability of the cells was determined by cell invasion assay, and **D** the protein levels of p-AKT and perilipin1 were detected by Western blotting. **E**–**G** After treated with actinomycin D (5 μg/ml), CHKα mRNA levels were measured by RT-qPCR, and the percentage of remaining mRNAs in the designated group were plotted. **H** RIP assay showing that DHRS2 can bind to CHKα mRNA in both OVCAR3 and HO-8910 cells. Data are shown as mean values ± S.D. of independent, triplicate experiments. The asterisks (*,**,***) indicate significant differences (*p* < 0.05, *p* < 0.01, *p* < 0.001, respectively).

To determine whether downregulation of CHKα contributes to the inhibitory effect of DHRS2 on OC progression, we re-expressed CHKα (Fig. 4A) or supplemented cells with choline, a substrate of CHKα, into DHRS2-transfected OC cells. We then measured cell proliferation and invasion. The results showed that DHRS2 expression attenuated the growth of OC cells (Fig. 4B). Compared with DHRS2-transfected cells, both forced CHKα expression and the addition of exogenous choline markedly reversed the inhibitory effect of DHRS2 (Fig. 4B). Similar alterations were observed in cell invasion assay results (Fig. 4C). AKT signaling has been shown to be involved in the promotive function of CHKα on the proliferation and invasion of cancer cells [28]. Moreover, we found that the phosphorylation level of AKT as well as perilipin1 expression was downregulated by DHRS2, whereas it was restored by CHKα re-expression or choline addition in both OVCAR3 and HO-8910 cells (Fig. 4D).

We further analyzed the expression of DHRS2 by using gene set enrichment analysis (GSEA) in cancer patients’ expression profiles obtained from multiple gene expression omnibus (GEO) databases. Interestingly, we found that DHRS2 expression levels showed a positive correlation with the RNA degradation pathway (Supplementary Fig. 2), which provided a clue that DHRS2 might affect the expression of CHKα through post-transcriptional pathways. Post-transcriptional regulations extensively affect the fate of mRNAs as well as the expression levels of the encoded proteins [29, 30]. To further determine whether DHRS2 affects the stability of *CHKα* mRNA, we used actinomycin D to block new RNA synthesis for 24 h, and subsequently detected *CHKα* mRNA level by RT-qPCR. The results showed that forced expression of DHRS2 reduced the half-life of *CHKα* mRNA in both OVCAR3 and HO-8910 cells (Fig. 4E, F). In contrast, DHRS2 depletion enhanced the stability of *CHKα* mRNA (Fig. 4G). Next, an RNA immunoprecipitation (RIP) assay was performed to determine whether DHRS2 regulates the stability of *CHKα* mRNA in a direct or indirect manner. Intringuing, we observed that DHRS2 can directly bind to *CHKα* mRNA in both OVCAR3 and HO-8910 cells (Fig. 4H), which suggests that DHRS2 might act as a potential RNA-binding protein (RBP) to control the degradation of *CHKα* mRNA.

Therefore, these results support several conclusions: (1) the downregulation of CHKα mediates the tumor-suppressive effect of DHRS2 on OC cells; (2) DHRS2 might directly bind to *CHKα* mRNA to promote its degradation, and consequently, decrease the protein level of CHKα.

### DHRS2 impairs tumor growth in vivo by interfering with the CHKα-AKT axis and choline metabolism

**Fig. 5:**
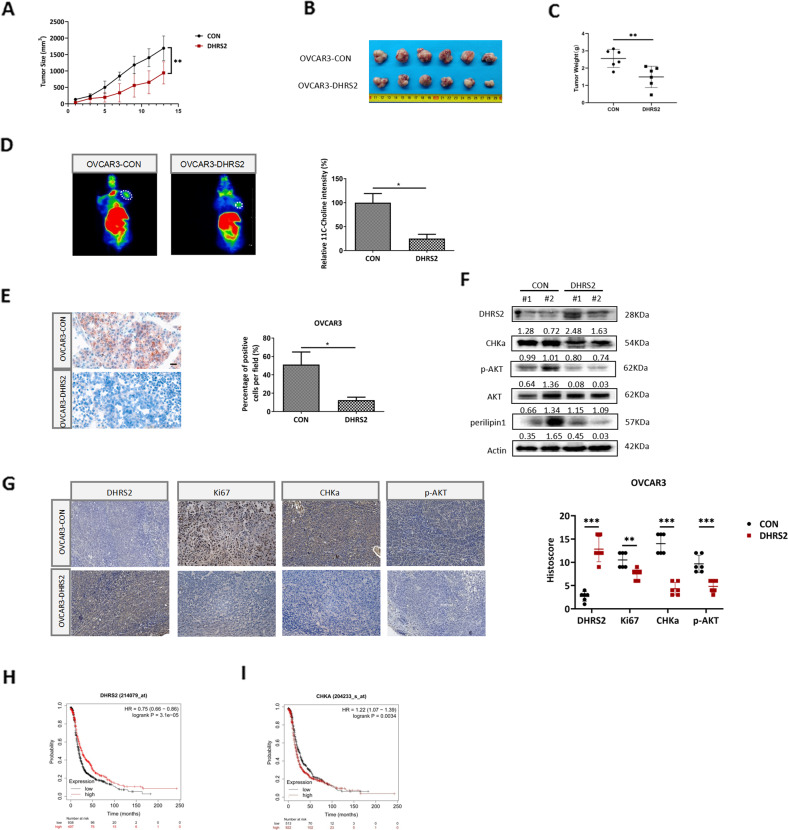
DHRS2 impairs tumor growth in vivo by interfering with the CHKα-AKT axis and choline metabolism. **A** The growth curve of OVCAR3-CON and OVCAR3-DHRS2 cells in vivo. Female BALB/c nu/nu mice were subcutaneously inoculated with OVCAR3-CON and OVCAR3-DHRS2 cells (*n* = 6 per group). Tumor volume was examined every other day and shown in the graph. **B** At the end of the experiment, the mice were sacrificed and the tumors were separated. **C** Tumor mass of each group was measured and shown in the graph. **D** ^11^C-Choline micro-PET/CT was performed and standardized uptake value (SUV) intensity was observed in the designated groups. **E** Representative images of Oil Red O staining in tumor tissues of the designated group. **F** The protein levels of DHRS2, CHKα, p-AKT, AKT, and perilipin1 in tumor tissues of the designated group. **G** Representative images of tumor sections in each group stained with indicated antibodies. Antibody staining is in brown and nuclear counter staining is in blue. Scatter diagram shows Histoscore for the indicated antibody staining in tumor samples. Survival analysis from TCGA dataset assessed by the Kaplan–Meier method for OC patients with high or low signature of **H** *DHRS2* and **I** *CHKα* genes. Data are shown as mean values ± S.D. of independent, triplicate experiments. The asterisks (*,**,***) indicate significant differences (*p* < 0.05, *p* < 0.01, *p* < 0.001, respectively).

To evaluate the effect of DHRS2 on OC tumors in vivo, we subcutaneously injected stable OVCAR3-CON and OVCAR3-DHRS2 cells, respectively, into BALB/C nude mice to generate xenograft tumor models. The mice in the OVCAR3-DHRS2 group showed delayed tumor growth relative to those in the OVCAR3-CON group (Fig. 5A). DHRS2 overexpression resulted in significant reduction of tumor volume and mass (Fig. 5B, C). In order to assess choline metabolic activity in xenograft tumors, ^11^C-choline micro-positron emission tomography was performed. The experimental results demonstrated that compared with OVCAR3-CON group, the tumor tissue in the OVCAR3-DHRS2 group displayed substantially reduced absorption of ^11^C-choline (Fig. 5D). This indicated that DHRS2 overexpression impaired choline uptake in tumors. Oil red O staining further illustrated that re-expression of DHRS2 resulted in a significant decrease of LD content in tumor tissues (Fig. 5E). Moreover, relative to mice in the OVCAR3-CON group, DHRS2 protein expression was enhanced; in contrast, CHKα, p-AKT, and perilipin1 protein levels were markedly reduced in tumor tissues of the OVCAR3-DHRS2 group (Fig. 5F). Immunohistochemistry (IHC) analysis supported this observation, and showed that DHRS2 overexpression substantially decreased Ki67 expression (Fig. 5G). We further investigated the association between DHRS2 and clinicopathological characteristics of OC by analyzing the data from TCGA and GEO datasets. We found that DHRS2 expression was markedly higher in normal tissues than that in OC tissues (*p* < 0.0001; Supplementary Fig. 3), while no significant difference was observed between different grade tumors (Supplementary Fig. 3). Survival analysis using the Kaplan–Meier method revealed that DHRS2 expression was positively correlated with the prognosis of OC patients (*p* < 0.001, Fig. 5H), whereas CHKα expression had a negative association with the overall survival of OC patients (*p* < 0.01, Fig. 5I). The expression of DHRS2 showed a negative association with that of CHKα, and CHKα expression positively correlated with perilipin1 (*p* < 0.05; Supplementary Fig. 4). Moreover, DHRS2 expression inversely correlated to AKT signaling (*p* < 0.05; Supplementary Fig. 4). Overall, our data support that DHRS2 impairs tumor growth in vivo by the interfering with CHKα-AKT axis and choline metabolism.

### DHRS2 hampers the invasion and metastasis of OC in vivo

**Fig. 6:**
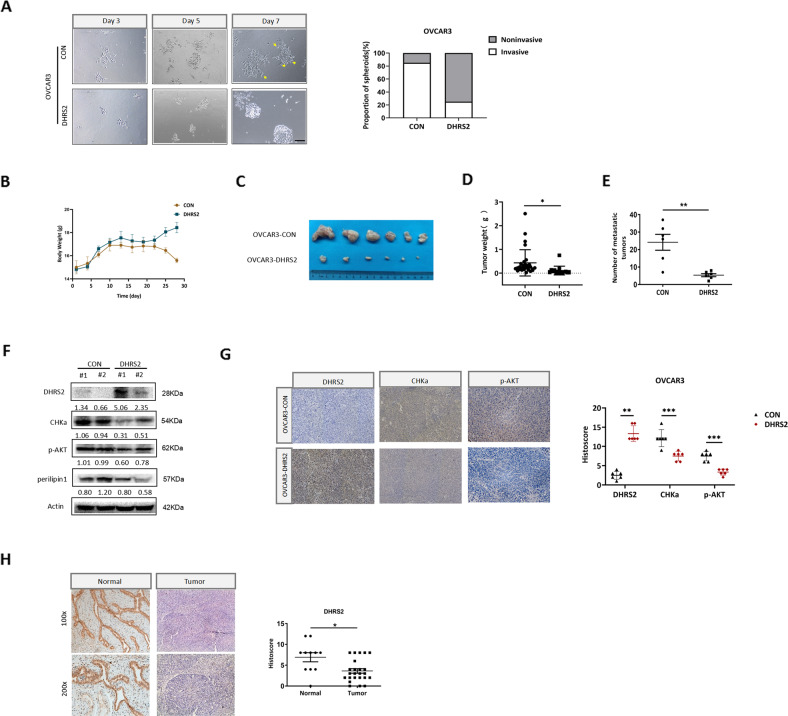
DHRS2 hampers the invasion and metastasis of OC cells. **A** Representative images of spheroids formed by OVCAR3 cells in Matrigel (left). The invasive spheroids were featured by scattered protrusions formed on the spheroid surface, as indicated by yellow arrows. Scale bar, 50 μm. Proportion of two spheroid types in OVCAR3-CON and OVCAR3-DHRS2 cells (right) (*p* < 0.05). Stable OVCAR3-CON and OVCAR3-DHRS2 cells were injected into the abdominal cavity, respectively (*n* = 6 per group). **B** During the experiment, the body weight of the mice was determined every 3 days and shown in the graph. **C** Representative intraperitoneal metastatic tumors in the designated group. **D**, **E** The amount and mass of the metastatic tumors were shown in the graph. **F** The protein levels of DHRS2, CHKα, p-AKT, and perilipin1 in tumor tissues of the designated group. **G** Representative images of tumor sections in each group stained with indicated antibodies. **H** The representative images of DHRS2 staining in OC tissues and non-tumor tissues. The scatter diagram shows Histoscore for DHRS2 staining in the designated samples. Data are shown as mean values ± S.D. of independent, triplicate experiments. The asterisks (*,**,***) indicate significant differences (*p* < 0.05, *p* < 0.01, *p* < 0.001, respectively).

We have demonstrated that DHRS2 inhibits the invasive capacity of OC cells in vitro. A 3D Matrigel cell culture model was further used to simulate the extracellular microenvironment and reemerge the process of cell adhesion and invasion. Invasive spheroids with scattered protrusions were observed in OVCAR3-CON cells, whereas OVCAR3-DHRS2 cells displayed relatively smooth edges with fewer protrusions (Fig. 6A). In order to explore the role of DHRS2 in tumor metastasis in vivo, we established an intraperitoneal metastatic animal model. Stable OVCAR3-CON and OVCAR3-DHRS2 cells were injected into the abdominal cavity, respectively (*n* = 6 for each group). Our results showed that both the amount and mass of abdominal-metastatic tumors were remarkably decreased in the OVCAR3-DHRS2 group (Fig. 6B–E). DHRS2 protein expression was upregulated, whereas CHKα, p-AKT, and perilipin1 expression levels were substantially decreased in the OVCAR3-DHRS2 group relative to those in the OVCAR3-CON group (Fig. 6F). Similar observations were acquired by H&E and IHC staining (Fig. 6G, Supplementary Fig. 5). These findings indicate that DHRS2 hampers the invasion and metastasis of OC through the inhibition of CHKα-AKT axis and choline metabolism.

Moreover, we examined DHRS2 protein expression in 24 cases of OC tissues and 11 cases of normal specimens, and significantly reduced DHRS2 expression was observed in OC tissues relative to normal controls (Fig. 6H). Pearson correlation analysis revealed that DHRS2 expression was inversely correlated with the distal metastasis of OC (*p* < 0.05, Supplementary Table 1). This further supports the idea that DHRS2 downregulation contributes to the metastatic progression in OC patients.

## Discussion

The deregulation of choline metabolism is intensively involved in the pathogenesis and development of cancers, and thus is considered to be one of the metabolic hallmarks of cancer [31–34]. Deregulation of choline metabolism contributes to tumorigenesis, progression, invasiveness, and therapeutic resistance [35, 36]. In brain cancers, the tCho content has been used as a biomarker for clinical diagnosis and grading, as well as for radiation therapy planning [37]. Choline metabolites PC and GPC have also been proposed as biomarkers of tumor progression, and an elevated PC/GPC ratio is consistently observed in aggressive cancers [38].

**Fig. 7:**
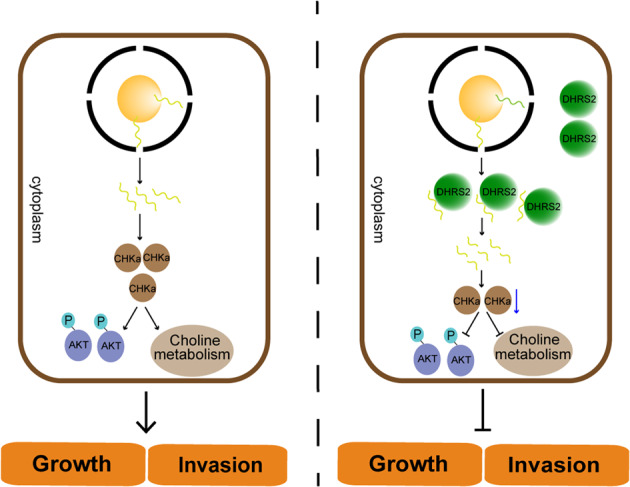
Schematic illustration of DHRS2 inhibiting OC growth and invasion by regulating choline metabolism. Left, CHKα mRNA is translated to protein, which promotes the activation of choline metabolism and AKT pathway, thereby promoting the growth and invasion of OC. Right, DHRS2 can directly bind to CHKα mRNA to accelerate its degradation. Correspondingly, CHKα protein expression is downregulated, resulting in the disruption of choline metabolism and AKT signaling. These effects lead to the inhibition of OC growth and metastasis. OC, ovarian cancer.

In this study, we demonstrated that DHRS2 inhibited the growth and metastasis of OC cells in vitro and in vivo (Fig. 7, Supplementary Fig. 6). The combination of transcriptome and metabolome analyses further suggested that the interruption of choline metabolism contributed to the tumor-suppressive function of DHRS2. Overexpression of DHRS2 substantially downregulated the PC/GPC ratio in OC cells. Thus, DHRS2 might impede OC progression by disrupting choline metabolism.

By screening the transcriptional profile of choline metabolic-associated genes, we found that *CHKα* was the most downregulated gene induced by DHRS2. CHK consists of three isoforms, CHKα1, CHKα2, and CHKβ. CHKα is highly expressed in a variety of cancers and has emerged as a promising therapeutic target for cancer [28, 39–42]. CHKα–CHKα dimers display substantially higher CHK activity relative to CHKβ–CHKβ homodimers or CHKα–CHKβ heterodimers. Thus the augmented CHK activity observed in cancer cells is most likely due to an increase in CHKα expression [39]. We further confirmed that DHRS2 downregulated CHKα expression, resulting in the disruption of choline metabolism as well as AKT signaling (Fig. 7). These actions led to the suppression of OC growth and metastasis. Studies have shown that the inhibition of the PI3K/AKT pathway reduced choline uptake in cancer cells, suggesting that this pathway affects choline transport [43]. Thereby, the inhibition of AKT signaling pathway induced by DHRS2 synergistically hampered choline metabolism. Another study also illustrated that in epithelial OC, CHKα depletion substantially impeded cell motility and invasiveness, while enhancing the therapeutic sensitivity. This observation further supported our conclusion that downregulation of CHKα mediates the inhibitory effect of DHRS2 on aggressive progression of OC (Fig. 7).

The events that regulate gene expression at the posttranscriptional level tightly control the storage, transport and turnover of mRNAs, and consequent protein expression and function [44]. In the present study, we found for the first time that DHRS2 directly binds to *CHKα* mRNA and promotes its degradation. This indicates that DHRS2 might be a novel RBP protein that participates in the post-transcriptional regulation.

Overall, we propose that activating DHRS2 to disrupt choline metabolism might be a novel target for OC therapy.

## Supplementary information

Supplementary materials and methods

Supplementary fig 1

Supplementary fig 2

Supplementary fig 3

Supplementary fig 4

Supplementary fig 5

Supplementary fig 6

Supplementary table 1

aj-checklist

## Data Availability

The omics data have been deposited to the repository of China National Center for Bioinformation (No. subPRO016469).
