# Supplementary material for: DHRS2 inhibits cell growth and metastasis in ovarian cancer by downregulation of CHKα to disrupt choline metabolism
Source: Cell Death Dis. 2022 Oct 3;13(10):845. doi: 10.1038/s41419-022-05291-w (PMC9530226; doi:10.1038/s41419-022-05291-w)
Supplement: Supplementary file 1 — Supplementary materials and methods [file 41419_2022_5291_MOESM1_ESM.docx]

Supplemental Materials and Methods

RNA extraction and quantitative real-time polymerase chain reaction (q-PCR)

Total RNA was extracted using the Nucleozol reagent (Macherey-nagel GmbH & Co, Düren, Germany) according to the protocol established by the manufacturer. Reverse transcriptional PCR was performed using the RevertAid First Strand cDNA Synthesis kit. The qPCR analysis was conducted in a 7500 Real Time PCR System (Applied BioSystems) using the SYBR Green PCR Supermix (Thermo Fisher Scientific). The PCR reaction conditions were 10 s at 95 °C followed by 40 cycles of 15 s at 95 °C and 60 s at 60°C.

Primers and shRNA sequence

shDHRS2 CCGGTCATCCCTGTGCTAGGCTTTCCTCGAGGAAAGCCTA

GCACAGGGATGATTTTTG

CHKα-F GATCCGAACAAGCTCAGAAAGAAAATG

CHKα-R CGGCTCGGGATGAACTGCTC

PDL1-F GGAAAGCGTGACAGTGAAATG

PDL1-R GATAGCCAAGGACAACCCTAAA

PLD2-F TCGATTTGCCGTTGCCTATTC

PLD2-R GGTCAAGAGACGGTTGAGGTA

GDPD5-F CTACAACCCTGAGCAGAT

GDPD5-R AACATACGGAGAGCACAT

GDPD6-F TTTCAAAATGCTGCAGGGTAAT

GDPD6-R ACCCACAAAGCAACAGTGTGTA

PLA2-F CAGCTACCAGTTCCACATCGT

PLA2-R CGGATTGCTTGTCACACTCAC

PLCL2-F CAGGGTGTGGCACATATAAATGA

PLCL2-R GGACCTCGGAACTGATCCTCTA

Western blotting

Cells were harvested and washed three times with PBS, then disrupted in IP lysis buffer (25 mM Tris-HCl, pH 7.4, 150 mM NaCl, 1% NP40, 1 mM EDTA, 5% glycerol; Thermo Scientific, MA, USA). Extracted proteins were quantified and separated by SDS-PAGE and transferred onto nylon membranes. After overnight incubation with the primary antibody, peroxidase-conjugated secondary antibodies were used to detect binding of primary antibodies. Visualization was performed by using the ChemiDoc XRS system with Image Lab software (Bio-Rad, CA, USA).

MTS assay

Cells were seeded in 96-well plates (2x103 cells per well). Then 10% CCK8 (Beyotime, China) was added at 0h, 24h, 48h, or 72h and cells were incubated at 37°C for another 2h. Absorption measurement was performed at 490 nm using a BioTek microplate reader (Beckman, Brea, CA, USA).

Colony formation assay

Cells were seeded into 6-well plates with 1×103 cells per well in triplicate and cultured for 10-14 days. Media were then discarded and cells were washed twice with PBS. The cells were fixed with methanol for 10 min, and then stained with crystal violet for an additional 10 min. Colonies containing more than 50 cells were counted by using the Photoshop software.

Cell invasion assay

The inside of an 8.0 μm-pore cell culture insert (BD Biosciences, Bedford, MA, USA) was coated with 0.75 mg/ml Matrigel (BD Biosciences) at 37 °C for 1 h. 8.0 × 104 cells in serum-free media were inoculated inside each chamber. The cells were treated with 10% FBS for 72 h and the chamber filter was fixed in 100% methanol and stained with crystal violet. The number of penetrated cells was counted under microscope and 3 independent assays were conducted.

Immunohistochemistry

The tumor tissue sections were deparaffinized using an environmentally friendly dewaxing agent (Solarbio, China) and rehydrated with ethanol-aqueous solutions of decreasing concentrations. For antigen retrieval, tissue sections were incubated in 10 mM sodium citrate buffer (pH = 6.0) for 20 min in a microwave oven. The endogenous peroxidase activity was removed by incubating with 3% hydrogen peroxide for 10 min and was blocked in normal donkey serum for 30 min. The primary antibodies were applied at 4°C overnight. Chromogen was developed using DAB (Zsgbbio, China) and counterstained with a hematoxylin staining kit. Immunohistochemical staining of these sections was evaluated based on all available tumor cells or epithelial cells meeting the typical morphological criteria as determined by 3 pathologists using the qualitative scale that is described in the literature. The number of cells stained was scored as 0 (no staining), 1 (< 1/3 positive cells), 2 (> 1/3 and < 2/3 positive cells) and 3 (> 2/3 positive cells). The intensity of staining ranged from 1 (weak) to 3 (strong). The immune reactive score was calculated by multiplying the percentage of positive cells and staining intensity.

Supplementary figure legends

Supplementary Fig.1 The protein levels of DHRS2 in OVCAR3, SKOV3, HO-8910, UWB1.289, UWB1.289+BRCA1, and PEO1 cells.

Supplementary Fig.2 GSEA analysis (#GSE9348) showed the different gene set between DHRS2low and DHRS2high. ES, enrichment score. NES, normalized enrichment score. *, p < 0.05.

Supplementary Fig.3 Clinical association of DHRS2 in OC. The data from the TCGA dataset showed DHRS2 expression in normal tissues and different OC tissue grades. ****, p < 0.0001. ns, no significance. The statistical difference of two groups was compared through the Wilcox test, significance difference of three groups was tested with Kruskal-Wallis test.

Supplementary Fig.4 Correlation analysis of DHRS2 expression and CHKa-AKT axis in OC. The expression correlation of two genes was analyzed with Spearman using the data from GEO datasets (GSE14407). (a) Correlation analysis of DHRS2 expression and CHKa. (b) Correlation analysis of CHKa expression and perilipin1. (c) Correlation analysis of DHRS2 expression and AKT signaling. The abscissa represents the expression distribution of the first gene, and the ordinate represents the expression distribution of the second gene. The density curve on the right represents the trend in distribution of the second gene, the upper density curve represents the trend in distribution of first gene expression. The value on the top represents the correlation p value, correlation coefficient and correlation calculation method.

Supplementary Fig.5 Images of metastatic tumor sections stained with hematoxylin-eosin (HE) in the designated groups.

Supplementary Fig.6 Original Western blotting data of the manuscript.

Supplementary table 1 Correlation analysis of DHRS2 downregulation with clinicopathologic characteristics of ovarian carcinoma patients
