# Supplementary figures and images for: DHRS2 inhibits cell growth and metastasis in ovarian cancer by downregulation of CHKα to disrupt choline metabolism
Source: Cell Death Dis. 2022 Oct 3;13(10):845. doi: 10.1038/s41419-022-05291-w (PMC9530226; doi:10.1038/s41419-022-05291-w)

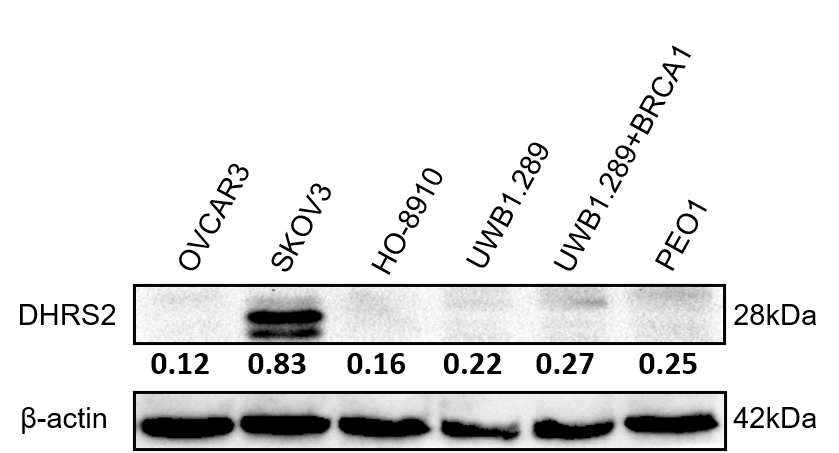

Supplement: Supplementary file 2 — Supplementary fig 1 [file 41419_2022_5291_MOESM2_ESM.tif]

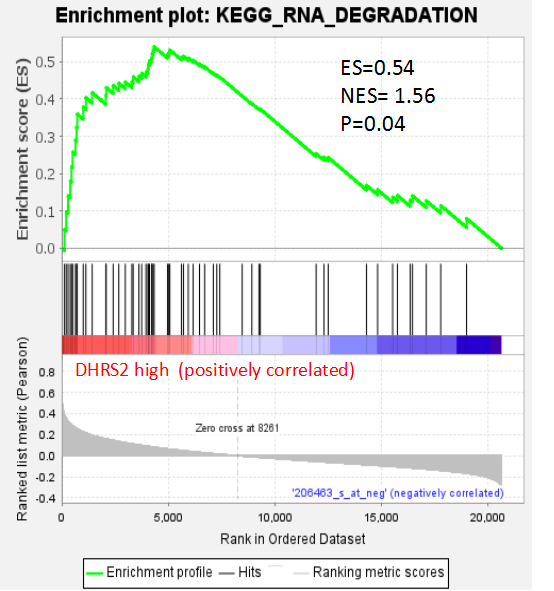

Supplement: Supplementary file 3 — Supplementary fig 2 [file 41419_2022_5291_MOESM3_ESM.tif]

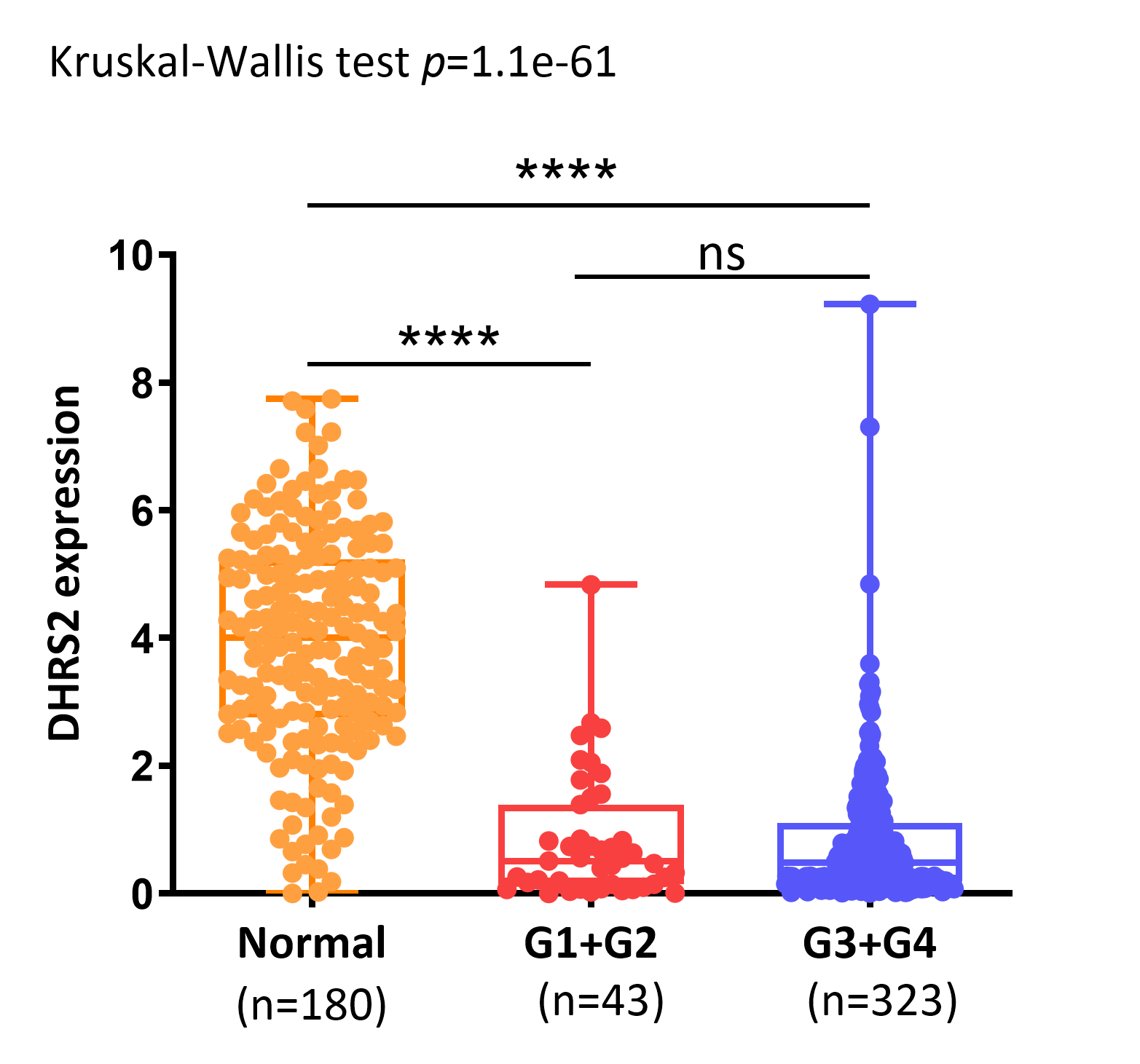

Supplement: Supplementary file 4 — Supplementary fig 3 [file 41419_2022_5291_MOESM4_ESM.tif]

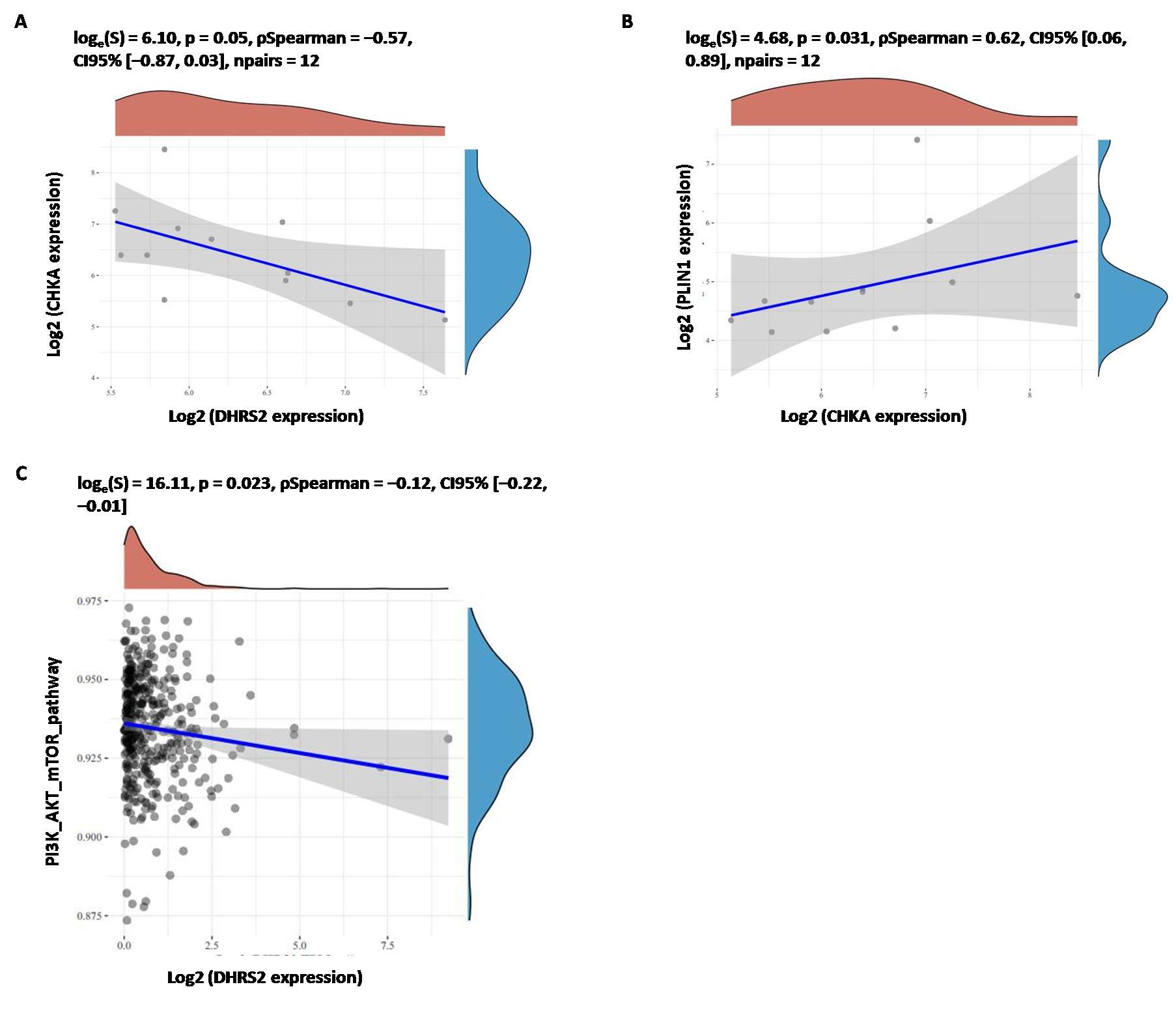

Supplement: Supplementary file 5 — Supplementary fig 4 [file 41419_2022_5291_MOESM5_ESM.tif]

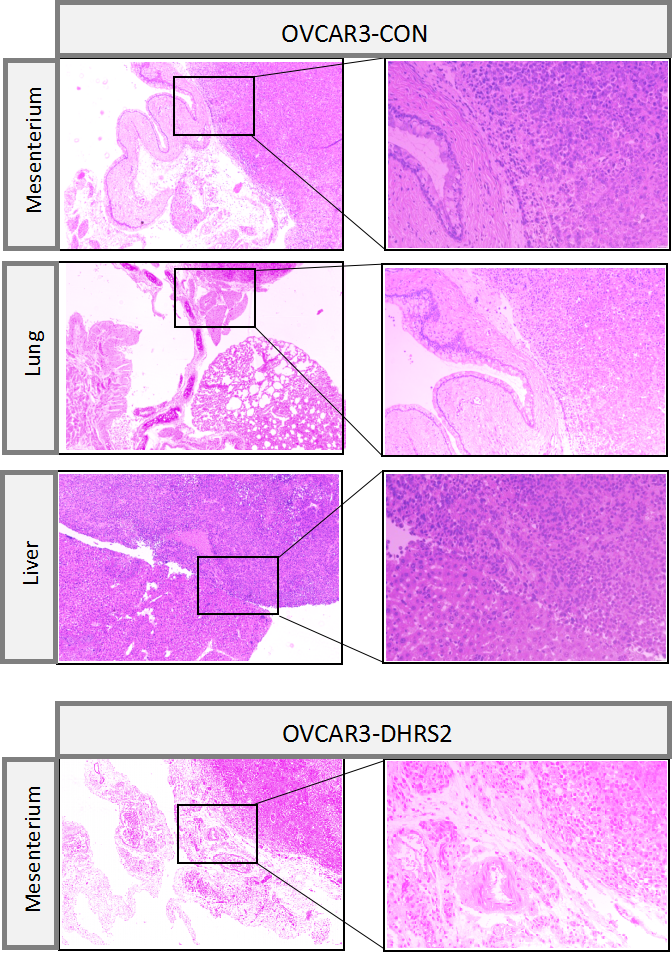

Supplement: Supplementary file 6 — Supplementary fig 5 [file 41419_2022_5291_MOESM6_ESM.tif]
