# Supplementary material for: DHRS2 inhibits cell growth and metastasis in ovarian cancer by downregulation of CHKα to disrupt choline metabolism
Source: Cell Death Dis. 2022 Oct 3;13(10):845. doi: 10.1038/s41419-022-05291-w (PMC9530226; doi:10.1038/s41419-022-05291-w)

Figure 1 A

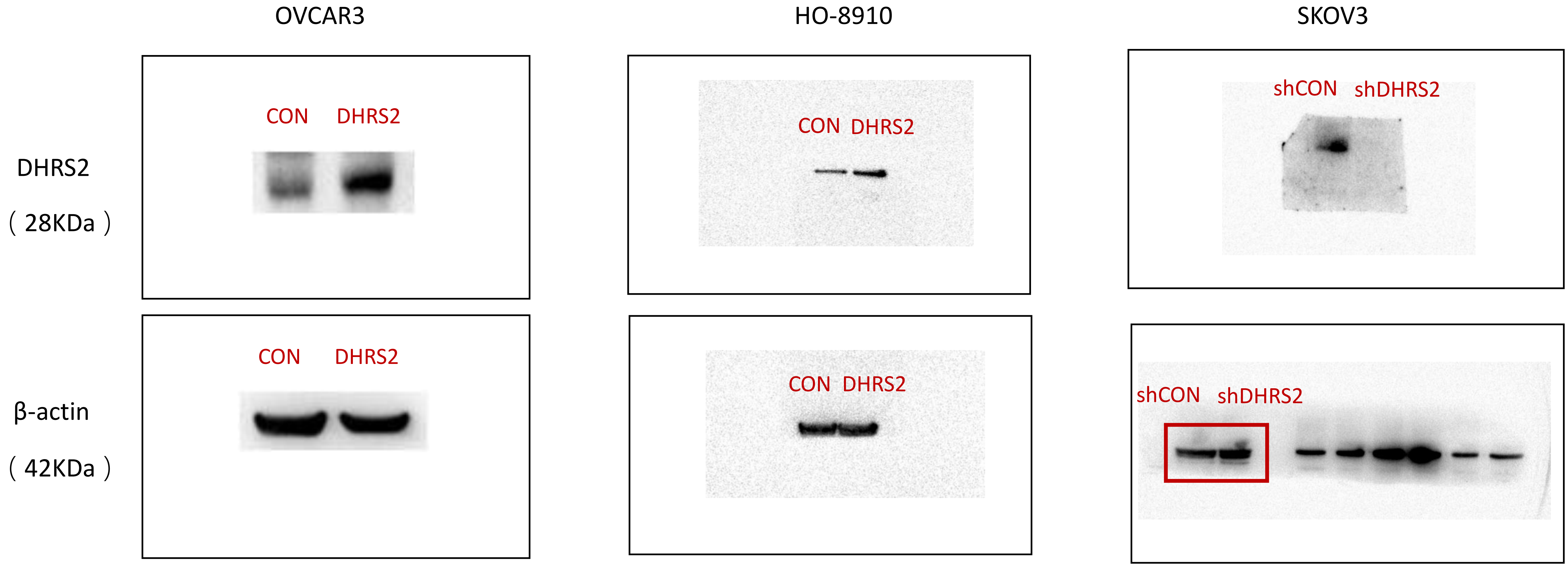

OVCAR3
HO-8910
SKOV3
DHRS2
(28KDa)
β-actin
(42KDa)
CON
DHRS2
CON
DHRS2
shCON
shDHRS2
CON
DHRS2
CON
DHRS2
shCON
shDHRS2

Figure 3 D

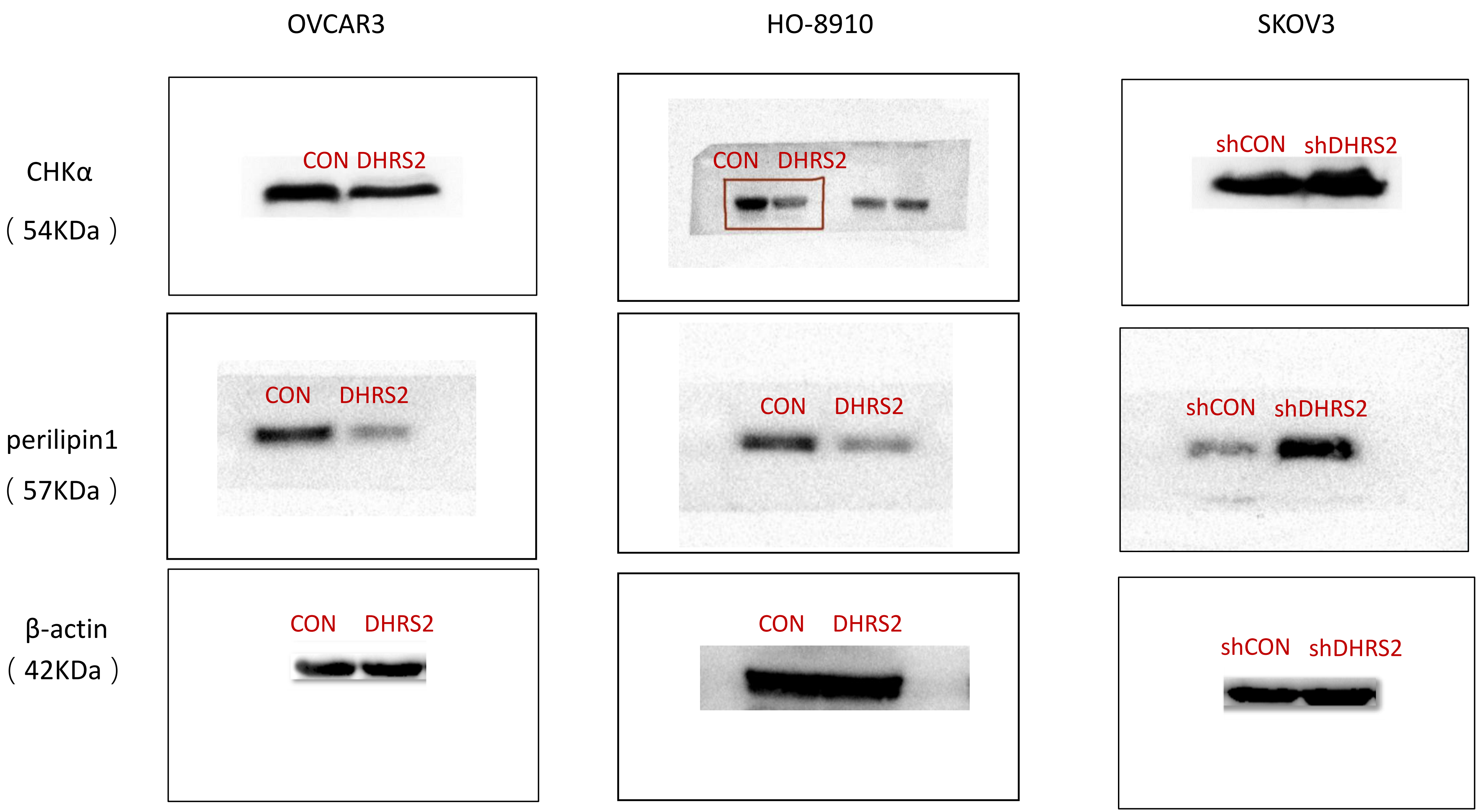
OVCAR3
HO-8910
SKOV3
CHKα
(54KDa)
perilipin1
(57KDa)
β-actin
(42KDa)
CON DHRS2
CON DHRS2
shCON shDHRS2
CON DHRS2
CON DHRS2
shCON shDHRS2
CON DHRS2
CON DHRS2
shCON shDHRS2

Figure 4 A

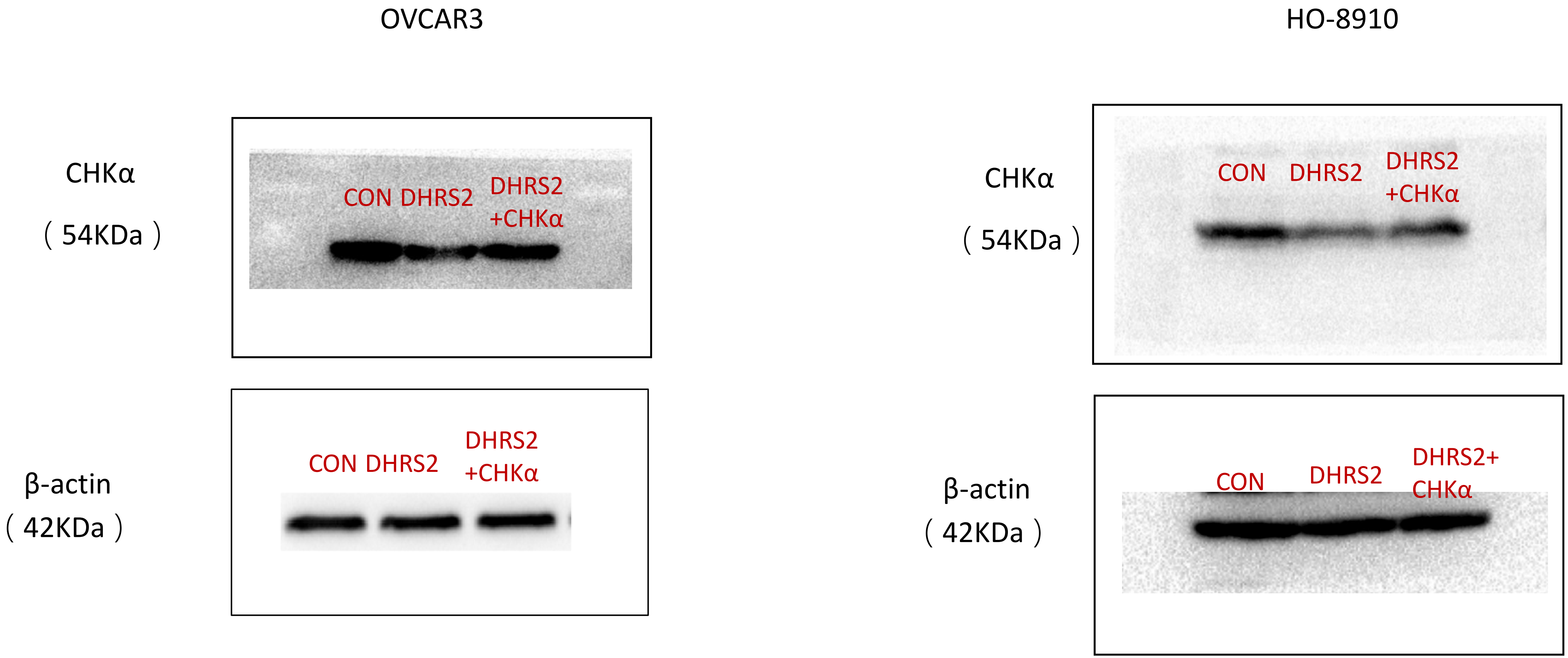
OVCAR3
HO-8910
CHKα
(54KDa)
CON DHRS2
DHRS2
+CHKα
β-actin
(42KDa)
CON DHRS2
DHRS2
+CHKα
CHKα
(54KDa)
CON
DHRS2
DHRS2
+CHKα
β-actin
(42KDa)
CON
DHRS2
DHRS2+
CHKα

Figure 4 D

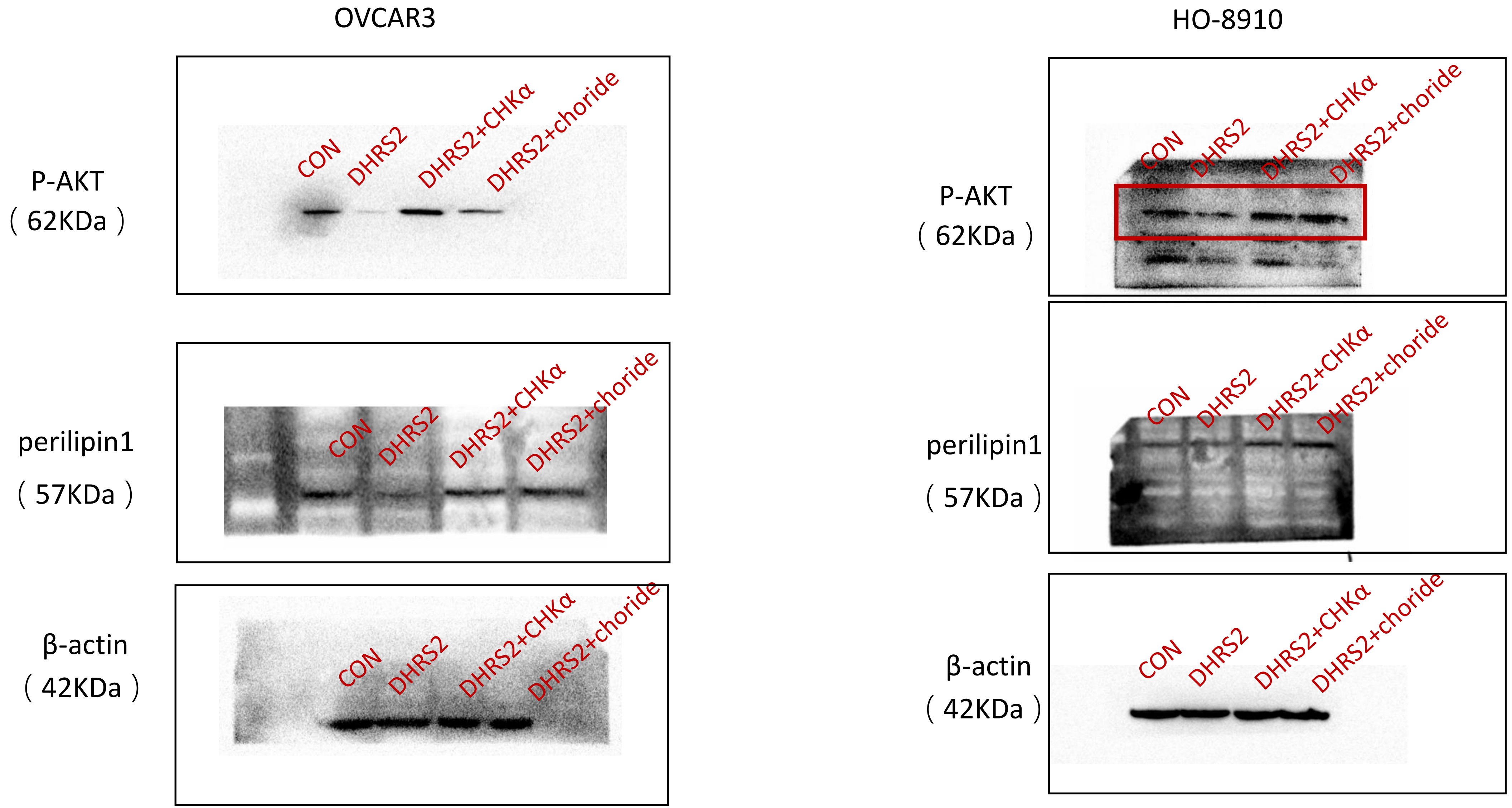
OVCAR3
HO-8910
P-AKT
(62KDa)
perilipin1
(57KDa)
β-actin
(42KDa)
CON
DHRS2
DHRS2+CHKα
DHRS2+choride

Figure 5 F

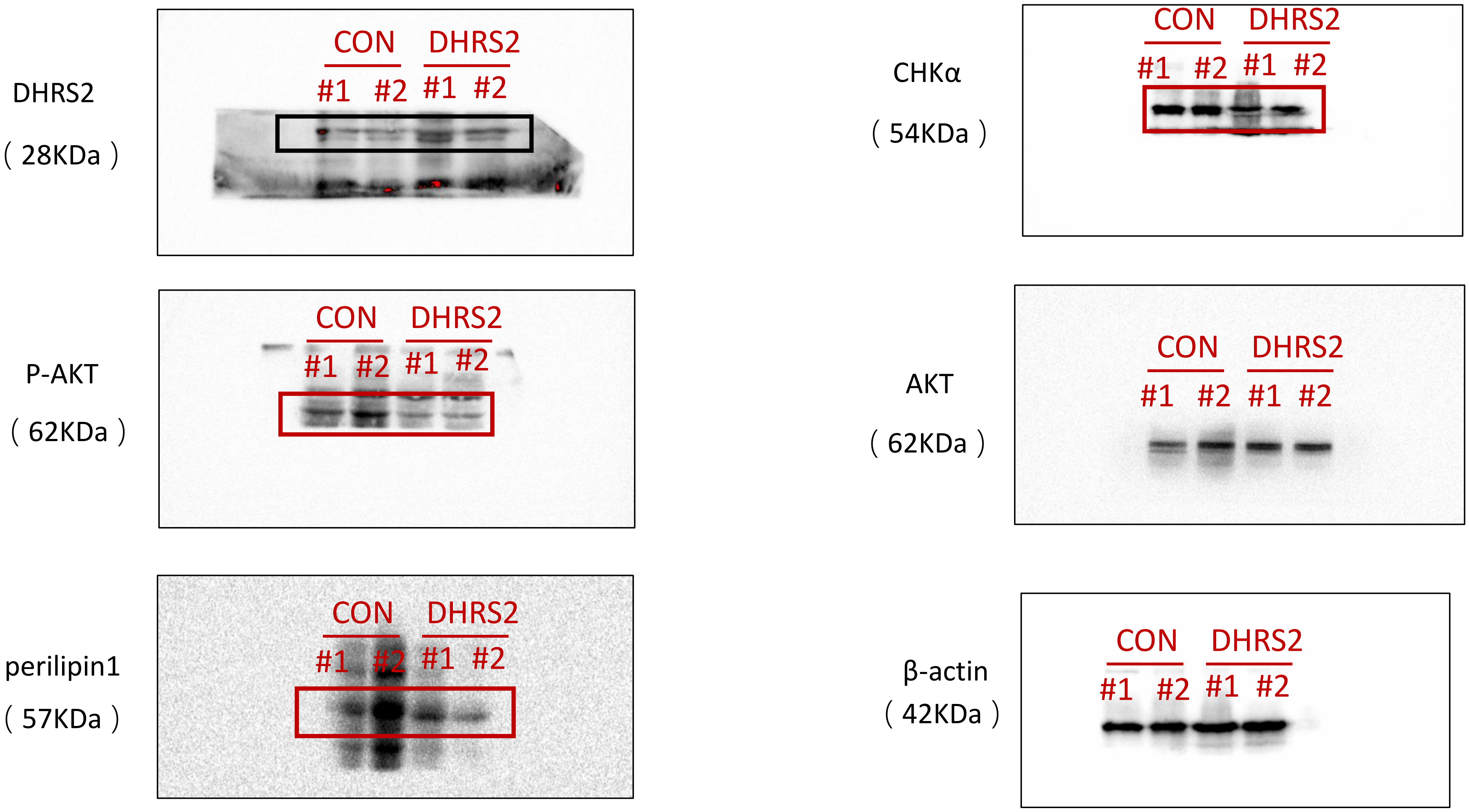
DHRS2
(28KDa)
CON
DHRS2
#1 #2 #1 #2
P-AKT
(62KDa)
CON
DHRS2
#1 #2 #1 #2
perilipin1
(57KDa)
CON
DHRS2
#1 #2 #1 #2
CHKα
(54KDa)
CON
DHRS2
#1 #2 #1 #2
AKT
(62KDa)
CON
DHRS2
#1 #2 #1 #2
β-actin
(42KDa)
CON
DHRS2
#1 #2 #1 #2

Figure 6 F

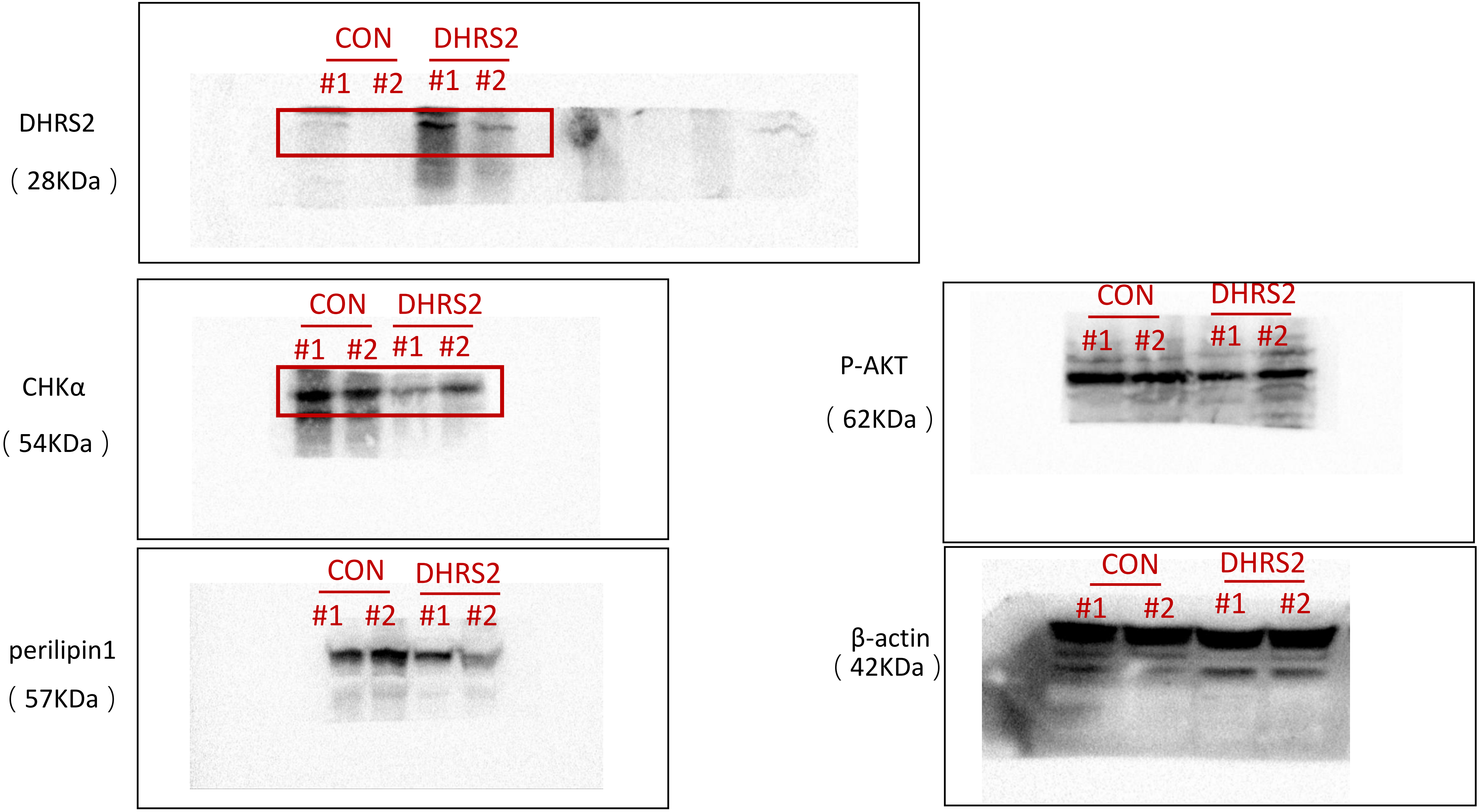

DHRS2
(28KDa)
CON
DHRS2
#1 #2 #1 #2
CHKα
(54KDa)
CON
DHRS2
#1 #2 #1 #2
perilipin1
(57KDa)
CON
DHRS2
#1 #2 #1 #2
P-AKT
(62KDa)
CON
DHRS2
#1 #2 #1 #2
β-actin
(42KDa)
CON
DHRS2
#1 #2 #1 #2

Supplement Figure 1

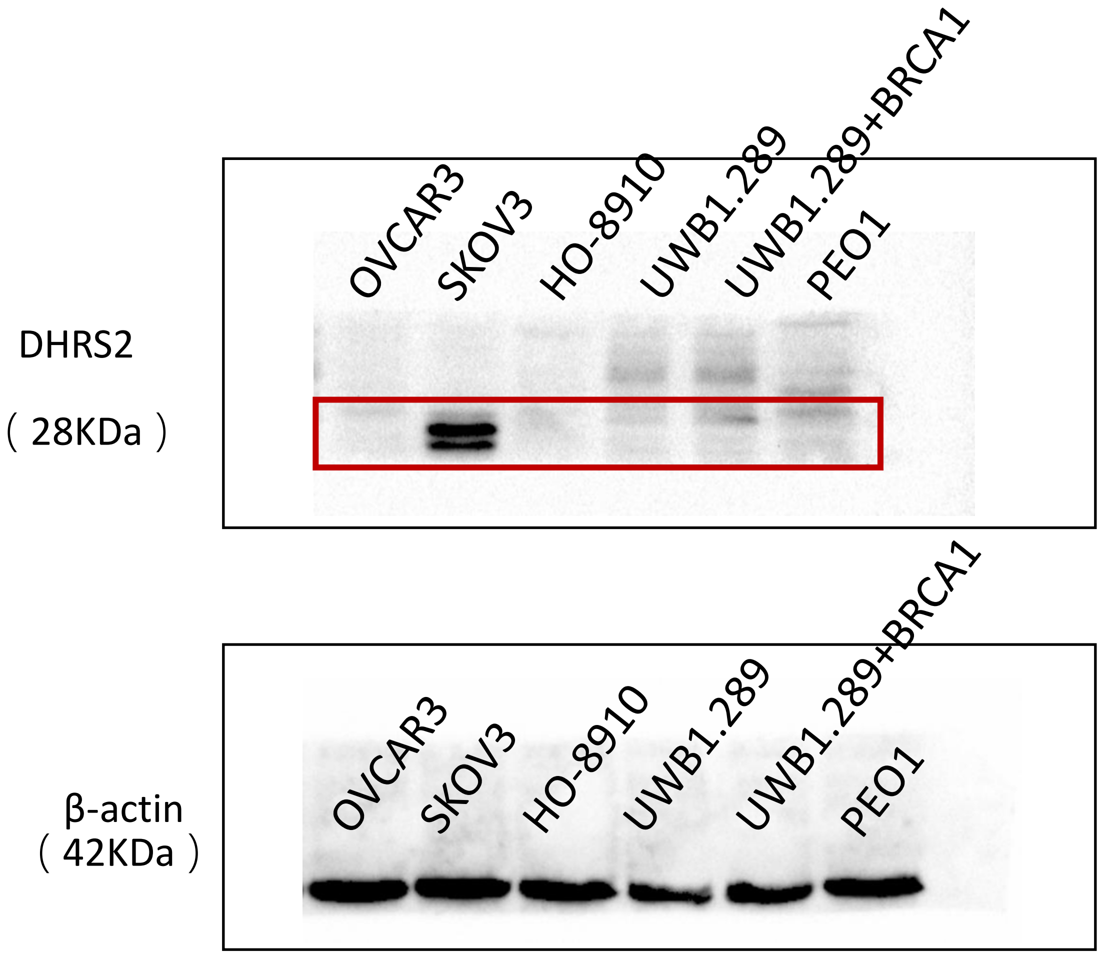

Supplement: Supplementary file 7 — Supplementary fig 6 [file 41419_2022_5291_MOESM7_ESM.pdf]
